# Retraction Note: The effect of uterine artery ligation in patients with central placenta pevia: a randomized controlled trial

**DOI:** 10.1186/s12884-023-05451-6

**Published:** 2023-02-27

**Authors:** Ahmad Sameer Sanad, Ahmad E. Mahran, Mahmoud Elmorsi Aboulfotouh, Hany Hassan Kamel, Hashem Fares Mohammed, Haitham A. Bahaa, Reham R. Elkateeb, Alaa Gamal Abdelazim, Mohamed Ahmed Zeen El-Din, Hossam El-Din Shawki

**Affiliations:** Obstetrics and Gynecology, Faculty of Medicine, Minia Maternity University Hospital, Minia University, PO Box 61111, Minia, Egypt

**Retraction Note: BMC Pregnancy Childbirth 18, 351 (2018)**

**https://doi.org/10.1186/s12884-018-1989-5**

The Editor has retracted this article. After publication, concerns were raised around p-values in Table 2, as well as the sample size and dates when the study was conducted. The authors provided raw data which did not fully correspond to the data presented in the article. They also proposed to correct the discrepancies, but some of the proposed corrections were also found to contain errors. The Editor has, therefore, lost confidence in the validity of the paper's findings. None of the authors agree to this retraction.

